# Supplementary material for: The Local Anaesthetic Procaine Prodrugs ProcCluster® and Procaine Hydrochloride Impair SARS-CoV-2 Replication and Egress In Vitro
Source: Int J Mol Sci. 2023 Sep 26;24(19):14584. doi: 10.3390/ijms241914584 (PMC10572566; doi:10.3390/ijms241914584)
Supplement: Supplementary file 1 [file ijms-24-14584-s001.zip › ijms-2550404-supplementary.pdf]

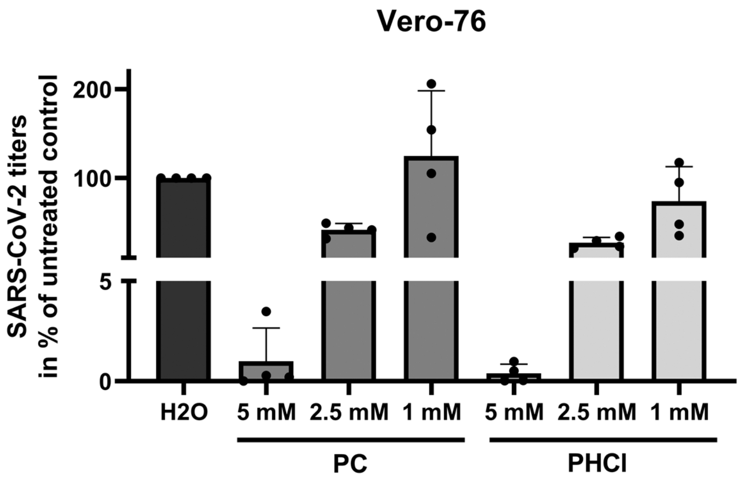

Supplementary Figure S1: Procaine treatment results in the inhibition of SARS-CoV-2 replication in Vero-76 cells. Cells were incubated with the indicated concentrations of PC or PHCl or solvent for 30 min prior to infection with MOI 0.5 of the early SARS-CoV-2 variant for 2 h. The inhibitors were present during infection and were added again after a washing step at 2 h p.i. Virus titres at 24 h p.i. were determined by plaque assay and are given in percent of the solvent-treated control. The mean (+SD) of three independent experiments with biological duplicates is depicted.

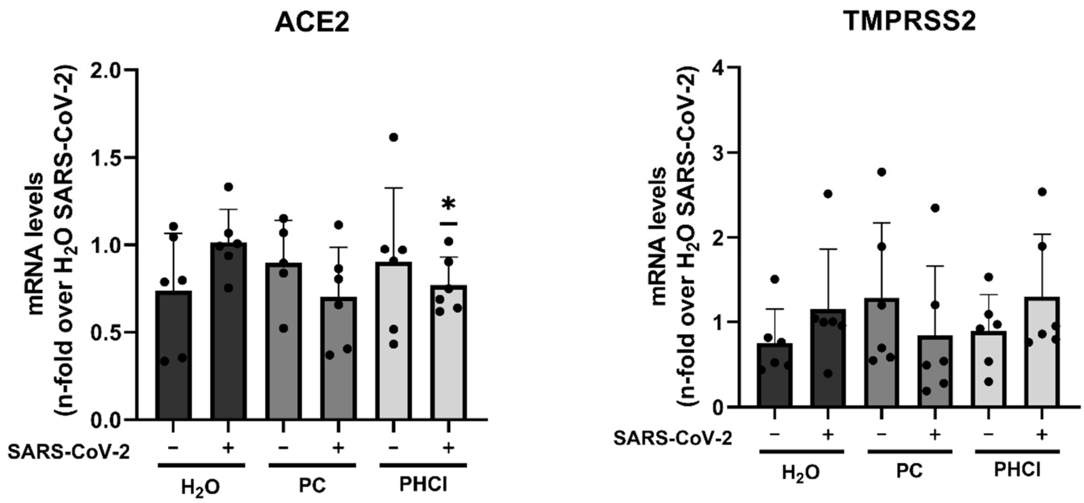

Supplementary Figure S2: The expression of ACE2 and TMPRSS2 is not affected by procaine treatment. Calu-3 cells were pre-treated with 2.5 mM PC or PHCl or solvent-control for 30 min prior to infection and then infected with 0.5 MOI of the early SARS-CoV-2 variant for 2 h in the presence of the inhibitor. The inhibitor was again added after a medium change and the cells were further incubated to 24 h p.i. RNA was extracted and the expression of ACE2 and TMPRSS2 was quantified by qRT-PCR. Solvent-treated infected samples were arbitrarily set to 1. The mean (+SD)

of three independent experiments with biological duplicates is depicted. Statistical significance was determined by one sample Wilcoxon test comparing to 1 (no statistical significance detected).

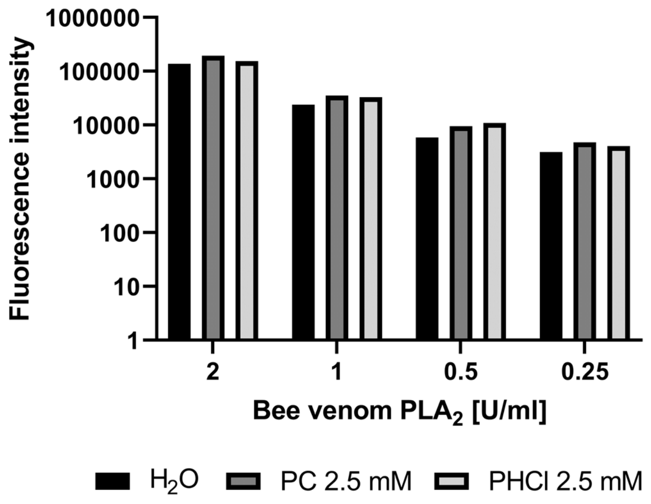

Supplementary Figure S3: Procaine does not inhibit the activity of phospholipase A2 (PLA_2) from bee venom. The activity of different amounts of bee venom PLA_2 in the presence of 2.5 mM PC or PHCl was measured using a fluorescent substrate (Excitation: 485 nm; Emission: 520 nm). Data from one experiment with technical duplicates is depicted.
